# Multiple Reaction Monitoring Mode Based Liquid Chromatography-Mass Spectrometry Method for Simultaneous Quantification of Brassinolide and Other Plant Hormones Involved in Abiotic Stresses

**DOI:** 10.1155/2016/7214087

**Published:** 2016-02-28

**Authors:** Deepak M. Kasote, Ritesh Ghosh, Jun Young Chung, Jonggeun Kim, Inhwan Bae, Hanhong Bae

**Affiliations:** ^1^School of Biotechnology, Yeungnam University, Gyeongsan, Gyeongbuk 712-749, Republic of Korea; ^2^Department of Orthopedic Surgery, School of Medicine, Ajou University, Suwon 442-749, Republic of Korea; ^3^College of Pharmacy, Chungang University, Seoul 156-756, Republic of Korea

## Abstract

Plant hormones are the key regulators of adaptive stress response. Abiotic stresses such as drought and salt are known to affect the growth and productivity of plants. It is well known that the levels of plant hormones such as zeatin (ZA), abscisic acid (ABA), salicylic acid (SA), jasmonic acid (JA), and brassinolide (BR) fluctuate upon abiotic stress exposure. At present, there is not any single suitable liquid chromatography-mass spectrometry (LC-MS) method for simultaneous analysis of BR and other plant hormones involved in abiotic stresses. In the present study, we developed a simple, sensitive, and rapid method for simultaneous analysis of five major plant hormones, ZA, ABA, JA, SA, and BR, which are directly or indirectly involved in drought and salt stresses. The optimized extraction procedure was simple and easy to use for simultaneous measurement of these plant hormones in* Arabidopsis thaliana*. The developed method is highly reproducible and can be adapted for simultaneous measurement of changes in plant hormones (ZA, ABA, JA, SA, and BR) in response to abiotic stresses in plants like* A. thaliana* and tomato.

## 1. Introduction

Plants are subjected to different biotic and abiotic stresses worldwide, including pathogens, heat, drought, and salinity, which severely affects their growth and productivity. Plant hormones are key regulators of adaptive stress response. It has been well established that plants normally accumulate various types of hormones upon exposure to drought [1, 2]. Abscisic acid (ABA) is a well-documented abiotic stress hormone that plays a major role in stress signaling, while jasmonic acid (JA) and salicylic acid (SA) are prominent regulators of biotic stress tolerance. Drought-mediated elevation of ABA is one of the first responses, which subsequently controls various physiological changes and triggers ABA inducible genes [1]. During drought stress, elevated accumulation of ABA results in closure of the stomatal opening to prevent water loss. In addition to ABA, hormones such as SA, JA, brassinolide (BR), and cytokinins (CKs) are involved in abiotic stress response, either in a synergistic or in antagonistic way [1]. Although ABA is the master regulator of stomatal closing, JA, BR, and SA have also been shown to play roles in stomatal closure under drought stress [2, 3]. Zeatin (ZA), a natural plant growth hormone that belongs to the CKs family, is involved in extensive crosstalk with ABA during stress adaptation [1]. Brassinosteroids, which are steroidal plant hormones, primarily engage in growth promotion as well as in various abiotic stresses including drought [4, 5]. It has been reported that exogenous application of BR might induce drought resistance in various plant species such as* Arabidopsis thaliana* [4, 6]. Similarly, SA is able to increase drought tolerance upon exogenous application [3, 7]. Moreover, the endogenous JA level was found to be upregulated in various plants under drought stress conditions [8, 9].

The measurement of endogenous hormonal levels in plants has received a great deal of interest owing to their potential role in signaling networks and molecular mechanisms. Consequently, several analytical methods have been adapted to measure plant hormones, including enzyme-linked immunosorbent assay (ELISA), high-performance liquid chromatography with diode-array and fluorometric detection (HPLC-DAD/FLD), liquid chromatography-mass spectrometry (LC-MS), and gas chromatography-mass spectrometry (GC-MS) [10, 11]. Among these, mass spectrometry (MS) methods have been found to provide high sensitivity and selectivity for analysis of plant hormones [12]. GC-MS is routinely applied for the simultaneous analysis of various hormones in plant tissues; however, it is relatively time consuming when compared to LC-MS [11]. Although several LC-MS methods have been developed for simultaneous analysis of multiple plant hormones [11–15], there is no any single suitable LC-MS method for simultaneous analysis of BR and other major hormones involved in drought stress. The probable reason behind this could be the concentration of BR or high matrix effect caused by other coeluting plant hormones.

**Figure 1:**
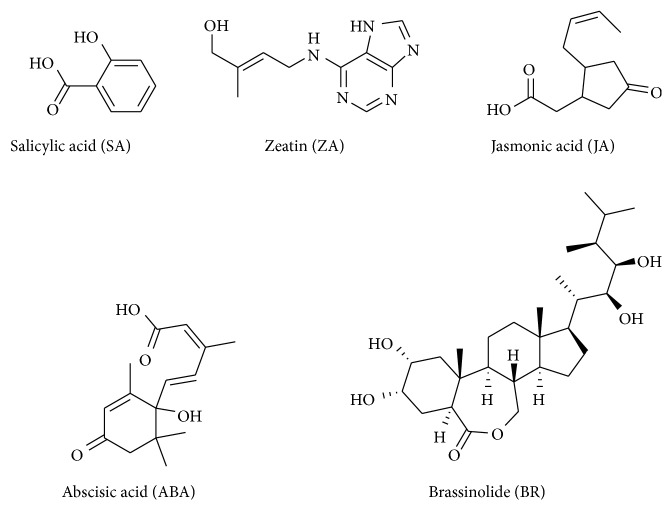
Structures of five plant hormones.

The present study was designed to develop a simple, rapid, and sensitive multiple reaction monitoring (MRM) mode based method that employed ultrafast liquid chromatography coupled tandem triple quadrupole mass spectrometry (UFLC-MS/MS) with electrospray ionization (ESI) for simultaneous analysis of BR and other plant hormones such as ZA, ABA, JA, and SA (chemical structures shown in Figure 1), which plays vital role in abiotic stress responses.

## 2. Materials and Methods

### 2.1. Chemicals

Acetonitrile, methanol, and water of HPLC grade were obtained from Fisher Scientific Korea Ltd. (Korea). Extrapure grades of isopropanol, acetic acid, and formic acid were obtained from Duksan Pure Chemicals (Korea). Additionally, (±)-abscisic acid (ABA), (±)-jasmonic acid (JA) and salicylic acid (SA) were purchased from Sigma-Aldrich (USA), while zeatin (ZA) and brassinolide (BR) were obtained from Santa Cruz Biotechnology (USA).

### 2.2. Stock and Working Solution Preparation

All plant hormone standards (2 mg of each standard) were dissolved in methanol (40 mL) to obtain stock solutions of 50 *μ*g mL^−1^, which were stored at −20°C until use. Working standard solution containing 1 *μ*g mL^−1^ of ZA, 2 *μ*g mL^−1^ of SA, and JA, 10 and 20 *μ*g mL^−1^ of ABA and BR, respectively, were prepared by combining the stock solutions of five plant hormones (200 *μ*L of ZA; 400 *μ*L of SA and JA; 1 mL of ABA; and 2 mL of BR) and adjusting the concentrations by dilution with methanol (6 mL).

### 2.3. Plant Growth Condition

Seeds of a model plant,* A. thaliana* ecotypes Columbia (Col-0), were sown in artificial soil. For synchronous germination, samples were subjected to coldness (4°C) and darkness for 2 days, after which they were grown under continuous light (~150 ± 10 *μ*mol m^−2^ s^−1^) at 22°C in a growth room. Water with nutrient solution was given to the plants in a regular interval for up to 18 days, after which it was withheld for 9 days, and after that drought treated samples (rosette leaves) were harvested and ground immediately in liquid nitrogen. We also simultaneously harvested 25-day-old plant samples (rosette leaves) grown normally without drought stress. Similarly, for salt stress, 20 days old* A. thaliana* were treated with 250 mM NaCl exogenously, and control plants were treated with normal water. The leaves were harvested after 20 h and immediately ground in liquid nitrogen. Fine powder was prepared by crushing leaves into the mortar and pestle.

### 2.4. Extraction Procedure

Stressed and normal plant samples (rosette leaves) were harvested and finely ground in liquid nitrogen with a mortar and pestle. Each sample was then weighed (50 mg) and transferred into 2 mL Eppendorf tubes. Next, working solution of standards (20 *μ*L) was added to each sample. The samples were then extracted using 9.8 mL of MA. After vortexing, samples were centrifuged at 13,000 ×g for 5 min at 4°C. The supernatant was then collected (1 mL) and filtered through a 0.45 *μ*M nylon syringe filter (Whatman, Korea), after which each sample (10 *μ*L) was injected into a UFLC-MS/MS system for hormonal analysis.

### 2.5. Optimization of UFLC-MS/MS Conditions

LC-MS/MS analysis was conducted using UFLC system coupled to a triple quadrupole mass spectrometer (LC-MS-8040, Shimadzu, Japan). The separation of samples was achieved on an ACE UltraCore 2.5 SuperC18 (150 × 4.6 mm) column. The LC conditions were optimized as follows: solvent A was 0.1% formic acid in water, and solvent B was 100% acetonitrile. The gradient program for pump B was as follows: 0.01–2 min, 0–40%; 2–5 min, 40–60%; 5–13 min, 100%; and 13–15 min, 20%. The flow rate was set to 0.5 mL min^−1^ and the column temperature was set at 40°C.

The mass spectra were acquired in both positive and negative mode using electrospray ionization and quantification of all analytes was carried out in MRM mode. The optimized MRM parameters for all standards are summarized in Table 1. The other operating parameters were as follows: nebulizer gas flow, 3 L min^−1^; drying gas flow, 15 L min^−1^; desolvation line (DL) temperature, 250°C; and heat block temperature, 400°C. LabSolutions software (Shimadzu) was used to control the instruments as well as acquire and process the data.

### 2.6. Calibration Curve and Method Validation

To estimate the coefficient of determination (*r* ^2^), calibration curves were prepared at concentrations ranging from 5 to 60 ng mL^−1^ for ZA, 10 to 120 ng mL^−1^ for SA and JA, and from 50 to 600 ng mL^−1^ and 100 to 1200 ng mL^−1^ for ABA and BR, respectively. Calibration curves were constructed by plotting the obtained peak areas of analytes* versus* their concentrations. Instrumental LOD and LOQ were determined for each standard from a chromatogram of standard solutions based on signal to noise (S/N) ratios of 3 : 1 and 10 : 1, respectively [16].

The method was further validated for precision, accuracy, and matrix effect. The precision was evaluated by three repeated injections at lower concentration (LLOQ) of a working solution of standards. The % RSD values of *R*_*t*_ and peak areas were then calculated [17]. The accuracy of the method was measured by calculating the percent recovery of each standard plant hormone using the standard addition method. Known amounts of each standard were added to a prequantified sample solution and the amounts of respective standard were estimated by measuring the peak area ratios and fitting these values to the straight line equation of the calibration curve [18]. In addition, matrix induced SSE was assessed by comparison of the average area of matrix-matched standards with the average area of corresponding neat solvent standards [19].

### 2.7. Statistical Analysis

Statistical evaluation of data was carried out using Microsoft Excel 2010. Significance was determined by student's *t*-test.

## 3. Results and Discussion

### 3.1. Optimization of UFLC-MS and UFLC-MS/MS Conditions

In this study, determination of mass spectrum scanning mode, retention times (*R*_*t*_), precursor, and product ions for each standard was carried out manually, and the remaining dwell time and CV values were tuned automatically. Among these, *R*_*t*_, precursor, and product ions are essential to confirm each analyte. Table 1 summarizes the parameters of the optimized MRM transitions. Positive scan mode was selected for ZA, ABA, and BR because they showed intense precursor and base ion peaks in this mode. ABA revealed that precursor ion at *m*/*z* 247 might be due to loss of the water moiety from its precursor ion ([M+H-H_2_O]^+^), whereas SA and JA showed the best maximum intensities for precursor and base ions in negative mode.

**Figure 2:**
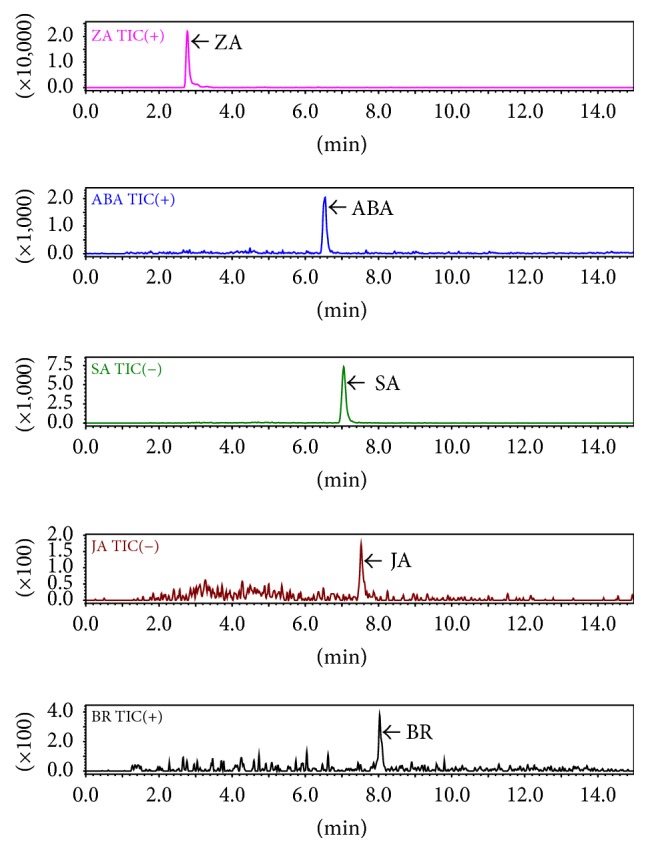
Typical MRM chromatograms of working solution of standard plant hormones [zeatin (ZA), abscisic acid (ABA), salicylic acid (SA), jasmonic acid (JA), and brassinolide (BR)].

A reserve phase Super C18 column was used for simultaneous separation of the five hormones. A typical MRM chromatogram of internal standards solution containing a mixture of ZA, ABA, SA, JA, and BR is shown in Figure 2. It has been reported that the addition of formic acid to the mobile phase greatly improves the peak sharpness and peak symmetry of acid plant hormones [17]. Hence, a binary gradient mobile phase consisting of 0.1% formic acid and acetonitrile was optimized to achieve sharp and high signal analytes for detection. The column oven temperature was set to 40°C to obtain symmetry of analyte peaks [20]. Dwell times of all MRM transitions were optimized to 100 ms to allow the collection of sufficient data points over each peak. The advantage of the current developed method is that it enables simultaneous separation of five plant hormones with a minimum flow rate and less run time.

### 3.2. Method Validation

Validation experiments were carried out as per guidance issued by the US FDA for Bioanalytical Method Validation (2001) [21]. For linearity analysis, the coefficient of determination (*r* ^2^) of each plant standard hormone was evaluated by plotting the peak area ratio versus the concentration in triplicate. *r* ^2^ value of each hormone is summarized Table 2. Generally, when the value of *r* ^2^ is 1 or very close to 1 (>0.99), it is considered to fit the regression line well. The resultant *r* ^2^ values for all other hormones were almost 1, which indicated good linearity within the considered concentration ranges [11]. To determine the sensitivity of this method, the limit of detection (LOD) and limit of quantification (LOQ) values for all plant hormones were calculated based on calibration curves in the matrix (Table 2). The precision of the UFLC-MS/MS method for all hormones is also documented in Table 2. The % RSDs (relative standard deviations) of the retention time and peak area of all standards were within the acceptable limits (<20% at the studied concentration), which confirms that the developed method is accurate and precise [22]. Similarly, the results of the percent recovery (% RE) of all hormones were also within an acceptable range (80–120%, Table 2). The matrix effect, which is generally defined as any change in the ionization process of an analyte due to a coeluting compound [23], is expressed as the % SSE (percent signal enhancement or suppression). The values of % SSE for all hormonal analytes are presented in Table 2. Evaluation of matrix effects during the quantitative analysis of compounds is an important aspect of assay validation, since it can cause ion suppression or enhancement of the analyte [24]. It has been reported that, when the % SSE value is equal to 100%, there are no matrix effects, while % SSE values less than 100% indicate suppression and those greater than 100% show enhancement of the ionization process [25]. Enhancement of ionization process was observed for ZA, ABA, and SA, whereas BR and JA showed ion suppression. To compensate for matrix effect and to improve the BR detection, a standard addition method was adopted for simultaneous quantification of plant hormones from fresh plant samples, in which plant samples spiked with known amounts of working solution of standards, and extracted later as per described above.

### 3.3. Optimization of Solvent Extraction Method and Validation

**Figure 3:**
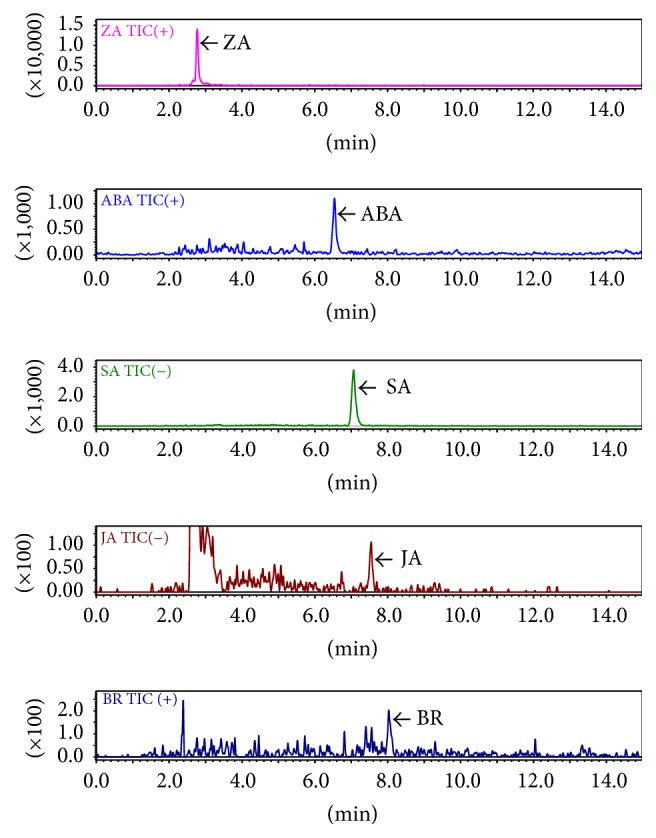
Typical MRM chromatograms of zeatin (ZA), abscisic acid (ABA), salicylic acid (SA), jasmonic acid (JA), and brassinolide (BR) in the crude extract of* A. thaliana* rosette leaves.

**Figure 4:**
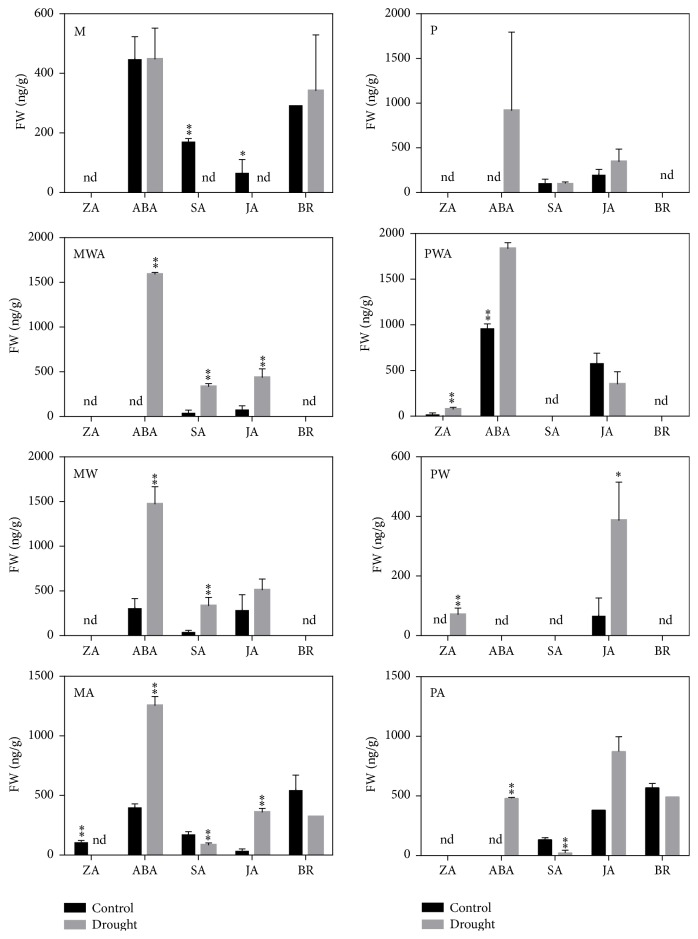
Effect of solvents on plant hormone extraction. Concentration of endogenous plant hormones (ZA, ABA, SA, JA, and BR) which were extracted by eight different solvents [P-100% isopropanol, M-100% methanol, PW (75 : 25, isopropanol : water), MW (75 : 25, methanol : water), PWA (75 : 24 : 1, isopropanol : water : acetic acid), MWA (75 : 24 : 1, methanol : water : acetic acid), PA (99 : 1, isopropanol : acetic acid), and MA (99 : 1, methanol : acetic acid)] from control and drought-stressed* A. thaliana* rosette leaves. “FW” represents fresh weight of plant sample. Asterisks denote a significant hormonal difference between control and drought-stressed plants and “nd” refers to not detected (*n* = 2/3, *t*-test, ^*∗*^ *P* < 0.1, ^*∗∗*^ *P* < 0.05).

**Figure 5:**
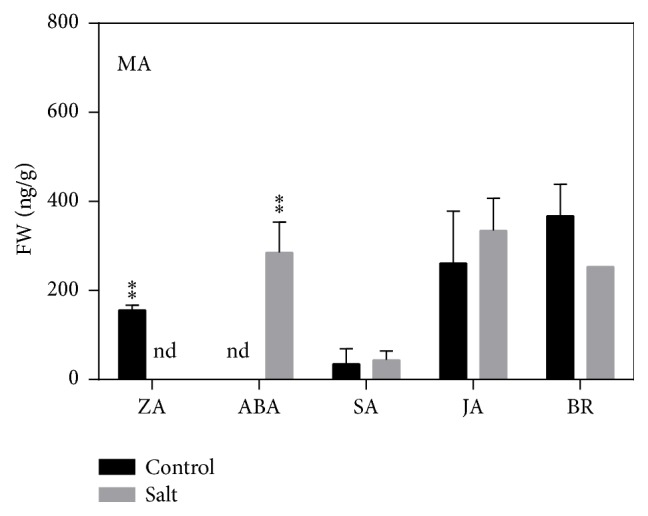
Hormonal levels from normal and salt-stressed* A. thaliana* rosette leaves samples after extraction using solvent methanol : acetic acid (99 : 1, v/v). “FW” represents fresh weight of plant sample. Asterisks indicate significant differences and “nd” refers to not detected (*n* = 2/3, *P* < 0.05, Student's *t*-test).

To date, organic solvent extraction has been the preferred method for extraction of hormones from plant samples. Several solvent extraction procedures have been developed using methanol, methanol/water mixture, acetone, acetone/water, propanol, propanol/water, and neutral or acid buffer [10, 11]. The polarity of the extraction solvent is generally chosen based on the optimum extraction of the target analytes. Methanol and isopropanol are the most widely used solvents for extraction of plant hormones [15]. Hence, in the present study, we conducted optimization experiments using methanol and isopropanol alone, as well as in combination with ultrapure water and acetic acid. Specifically, 100% isopropanol (P), 100% methanol (M), 75 : 25 isopropanol : water (PW), 75 : 25 methanol : water (MW), 75 : 24 : 1 isopropanol : water : acetic acid (PWA), 75 : 24 : 1 methanol : water : acetic acid (MWA) 99 : 1 methanol : acetic acid (MA) and 99 : 1 isopropanol:acetic acid (PA) were used for extraction. All five hormones were then separated and detected in the crude extract of* A. thaliana* rosette leaves. A typical MRM chromatogram of each hormone detected in the crude extract sample of* A. thaliana* rosette leaves is shown in Figure 3, and the extraction efficiency of each solvent is summarized in Figure 4. Extraction using MA showed the best recovery for almost all the studied hormones in both control and drought stressed samples, whereas the other extraction solvents showed comparatively low recoveries and were sometimes not enough to recover all the studied hormones. For validation of results, quantification hormones were also carried out in normal and salt stressed samples using MA solvent system (Figure 5).

## 4. Conclusions

We developed a simple, sensitive, and rapid LC-MS/MS method for simultaneous quantification of BR and other plant hormones such as ZA, ABA, JA, and SA involved in* A. thaliana* drought and salt stresses. This LC-MS/MS method was precise (% RSD < 15%) and accurate (% RE within 80–120%). In addition, reported solvent extraction procedure (99 : 1 methanol : acetic acid) was easy and less tedious for simultaneous isolation of these plant hormones. The above described procedure was successfully applied to isolate and quantify aforementioned plant hormones from* A. thaliana* that can also be used for other plants such as tomato (results of the plant hormone analysis from green tomato pericarp are summarized in supplementary material 1, in Supplementary Material available online at http://dx.doi.org/10.1155/2016/7214087).

## Supplementary Material

Hormonal levels in normal green tomato pericarp extracted using solvent methanol:acetic acid (99:1, v/v). (values are means of three independent samples ± standard error) [Zeatin (ZA), Abscisic acid (ABA), Salicylic acid (SA), Jasmonic acid (JA), and Brassinolide (BR)].

## Figures and Tables

**Table 1:** Optimized multiple reaction monitoring mode (MRM) conditions for the analysis of plant hormones.

Sr. number	Hormone	Mode (+/−)	*R*_*t*_ (min)	Precursor ion (*m*/*z*)	Product ion (*m*/*z*)	Dwell time (msec)	CV (eV)
1	Zeatin	+	2.78	220	119	100	−35
2	(±)-Abscisic acid	+	6.53	247	91	100	−35
3	Salicylic acid	−	7.05	137	93	100	35
4	(±)-Jasmonic acid	−	7.55	209	59	100	35
5	Brassinolide	+	8.03	481	95	100	−35

**Table 2:** Overview of validated parameters (*R*_*t*_, retention time; LOD, limit of detection; LOQ, limit of quantification; RE, recovery in percent; RSD, relative standard deviations; and SSE, signal suppression/enhancement).

Sr. number	Hormone	Coefficient of determination (*r* ^2^)	Precision (% RSD) at		RE (%)	SSE (%)	LOD (ng mL^−1^)	LOQ (ng mL^−1^)
			*R*_*t*_	Peak area				
1	Zeatin	0.989	0.63	10.4	83.8	105.7	0.26	0.80
2	(±)-Abscisic acid	0.984	0.31	6.7	92.7	103.7	0.49	1.48
3	Salicylic acid	0.998	0.14	0.08	93.1	104.0	0.04	0.13
4	(±)-Jasmonic acid	0.999	0.15	5.3	82.6	88.01	0.35	1.05
5	Brassinolide	0.990	0.12	19.9	87.5	96.2	0.29	0.87
