# Supplementary material for: Multiple Reaction Monitoring Mode Based Liquid Chromatography-Mass Spectrometry Method for Simultaneous Quantification of Brassinolide and Other Plant Hormones Involved in Abiotic Stresses
Source: Int J Anal Chem. 2016 Feb 28;2016:7214087. doi: 10.1155/2016/7214087 (PMC4789037; doi:10.1155/2016/7214087)
Supplement: Supplementary file 1 — Hormonal levels in normal green tomato pericarp extracted using solvent methanol:acetic acid (99:1, v/v). (values are means of three independent samples ± standard error) [Zeatin (ZA), Abscisic acid (ABA), Salicylic acid (SA), Jasmonic acid (JA), and Brassinolide (BR)]. [file 7214087.f1.doc]

**Supplementary file 1.**

**Hormonal levels in normal green tomato pericarp extracted using solvent methanol:acetic acid (99:1, v/v).** (values are means of three independent samples ± standard error) [Zeatin (ZA), Abscisic acid (ABA), Salicylic acid (SA), Jasmonic acid (JA), and Brassinolide (BR)].
